# Chaya Leaf: A Promising Approach for Diabetes Management

**DOI:** 10.3390/ph18091242

**Published:** 2025-08-22

**Authors:** Fabiola Curiel Ayala, Francisco Ignacio García Rodríguez, Sandra N. Jimenez-Garcia, Lina Garcia-Mier

**Affiliations:** 1 División de Ciencias de la Salud, Universidad del Valle de México, Campus Querétaro, Blvd. Juriquilla No. 1000, Santa Rosa Jáuregui, Santiago de Querétaro C.P. 76230, QRO, Mexico; fabiola.curiel@uvmnet.edu (F.C.A.); a110167380@my.uvm.edu.mx (F.I.G.R.); 2 Departamento de Enfermería y Obstetricia, División de Ciencias de la Salud e Ingeniería, Campus Celaya-Salvatierra, Universidad de Guanajuato, Av. Mutualismo Esq. Prolongación Río Lerma S/N, Celaya C.P. 38060, GTO, Mexico; sandra_neli_j@hotmail.com

**Keywords:** chaya leaf, diabetes, hypoglycemic effects, traditional medicine

## Abstract

Chaya leaf has long been used in folk medicine and is gaining scientific interest for its potential role in diabetes management. Recent research indicates that chaya leaf may help to regulate glucose, enhance insulin secretion, and reduce related complications, primarily due to the presence of bioactive compounds such as polyphenols and flavonoids. These compounds are believed to enhance insulin sensitivity and offer protection against oxidative stress, a key contributor to diabetes-related complications. Chaya extracts, particularly methanolic and aqueous forms, have shown anti-diabetic effects in animal models, lowering blood glucose, cholesterol, and triglycerides and reducing inflammation; their bioactive compounds, like quercetin, rutin, and ferulic acid, may enhance the insulin response, reduce inflammation, and improve antioxidant activity. Some studies warn of potential interactions with metformin. This review compiles findings from the past five years, drawing from databases such as PubMed, SciELO, ScienceDirect, Dialnet, Web of Science, and Google Scholar. It highlights chaya’s phytochemical profile, explores proposed anti-diabetic mechanisms, and summarizes evidence from in vivo, in vitro, and clinical studies. The results indicate that adding chaya leaf to the diet may help people with diabetes as a complementary therapy to conventional treatment; nonetheless, further clinical studies are required to comprehend the exact mechanisms and define specific usage instructions. Further investigation into the specific types of compounds present in chaya, their effective dosages, and their safety in human populations is essential to support its integration into medical practice.

## 1. Introduction

There is rising interest in investigating and recognizing sources like medicinal plants that have important phytochemicals and superior nutritional content compared to processed foods [1,2,3]. These sources could be used to create medications or food items that might aid in the prevention or management of transmissible chronic illnesses. Non-communicable diseases (NCDs) are health conditions that develop from interactions among factors and external influences, like the environment and physiology, which need continuous attention and management and could result in disability or early mortality [4]. Key NCD categories encompass cancer, diabetes, heart diseases, and chronic respiratory disorders. NCD-related fatalities represented 71 percent of deaths and comprised 14 of the leading causes of death in the Americas in 2019 [4,5]. In the first half of 2021, in Mexico, over 1.5 million cases of non-communicable diseases were reported. There has been a rise in people using plants, which have been crucial in health for centuries, mostly to treat injuries or illnesses [6,7,8,9]. For a plant to be labeled “medicinal”, it must show scientifically verified effects. Mexico boasts a variety of plants, totaling around 4500 species; however, only a small fraction of them, roughly 5%, have undergone pharmacological assessment [10]. One such plant that remains underexplored from this standpoint is chaya, which has been gaining attention for its health benefits, including blood sugar regulation [11,12]. Despite its properties, its application currently relies on empirical knowledge passed down through generations via oral communication, online publications, and recipe books. Knowledge about the bioactive compounds responsible for its health benefits, as well as whether there may be toxic or hazardous compounds in the plant, remains absent [13,14]. For this reason, the main aim of this review is to offer insights into the existing understanding of chaya’s role in managing diabetes and to delve into future research avenues, as well to promote the incorporation of foods that have been ancestrally used as therapeutic alternatives. Mechanisms of action, involving bioactive substances, as well as in vivo and in vitro studies, are summarized. Most of the details regarding the use of chaya to treat diabetes were mainly gathered from the last five years. We also included relevant information from earlier years. To conduct this review on chaya, a comprehensive search was performed across several scientific databases, including PubMed, Scielo, Science Direct, Dialnet, Web of Science, and Google Scholar. Keywords used in the search included “chaya”, “*Cnidoscolus chayamansa*”, “chaya leaf”, “diabetes”, and “*Cnidoscolus aconitifolius*”. First, Google Scholar was explored to determine whether the required information was available, where an initial number of 2310 articles were obtained. Then, the other databases were used. Articles were selected based on their relevance to the nutritional, pharmacological, and ethnobotanical aspects of chaya. Studies in English and Spanish were included. Duplicate, non-scientific, thesis, and popular articles were excluded. After screening the titles and abstracts, a total of 37 articles were selected for detailed review. The manuscripts were initially chosen based on their titles, before moving on to the abstracts and conducting an analysis of the text to confirm that they contained relevant information related to the subjects discussed in this review. The inclusion and exclusion of articles in this review focused on selecting studies that addressed key aspects of diabetes mellitus, as well as the implications of metabolites for the anti-diabetic effects of chaya. We performed the identification, screening, and selection according to the criteria stated previously.

## 2. Botanical and Ethnobotanical Information of *C. chayamansa*

**Figure 1:**
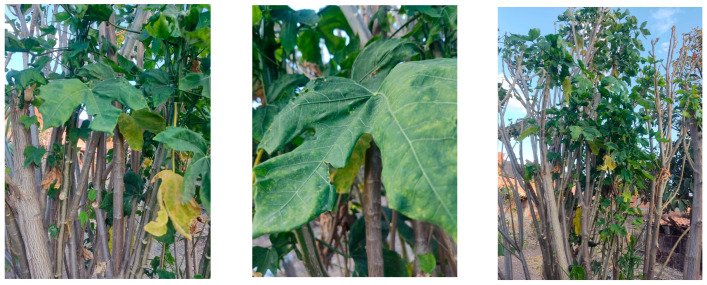
Chaya plant.

The plant known as chaya is also recognized by the names *Cnidoscolus chayamansa* McVaugh and *Cnidoscolus aconitifolius* (Mill) [12,15]. It is also referred to as “tree spinach”, or “quelite” [16]. It belongs to the *Euphorbiaceae* family and the genus *Cnidoscolus* spp. (Figure 1), which are distinguished by the number of lobes and the shape of their leaves. In the case of chaya, its leaves have three lobes connected by a petiole to the main stem. The plant can reach heights of 5 to 6 m based on the quality of the soil and its environment. It is native to the Yucatán Peninsula and is now grown in regions like Hidalgo and San Luis Potosí in Mexico. It is also being cultivated in Belize and Guatemala, as well as in the United States and Africa [12,15,16,17].

The chaya plant, or *Cnidoscolus chayamansa*, is commonly referred to as “chaya” or tree spinach, originating from the Maya regions in the Americas. It is a variety of the *Cnidoscolus aconitifolius* plant due to its lack of stinging hairs, which can cause skin irritation during harvesting. *C. chayamansa* is a cultivated, spineless (non-stinging) version of *C. aconitifolius*, selected for safer handling and easier consumption. It is not a separate species but a domesticated variant developed to be more suitable for human handling and food use. It is often grown in home gardens and used in cooking. When cooked as a vegetable dish for consumption purposes, chaya is rich in vitamin C content, along with beta carotene, proteins, calcium, phosphorus, iron, and vitamins, including riboflavin and niacin [12,15,18].

Throughout history, animals like livestock and horses have been given chaya as a food source. Historical records from the Maya civilization indicate that it was also consumed by humans due to its nutritional value [16]. Chaya stands out for its protein content exceeding 5%, containing seven of the nine essential amino acids (leucine, isoleucine, valine, lysine, methionine, phenylalanine, and threonine), and it has crude fiber content higher than 2% [12,19]. For this reason, people incorporate chaya leaves into their diets or consume them as an infusion. It is recommended to subject the leaves to heat treatment before consumption due to the presence of cyanogenic glycosides—compounds that are harmful to living beings but necessary for the plant’s defense against predators [16,20]. Cyanogenic glycosides release cyanide when the plant is damaged. In chaya, the main cyanogenic compound is linamarin (as found in cassava), but this is reduced to a safe level when the plant is cooked. The nutritional value of the plant may vary according to environmental fluctuations, soil conditions, biotic factors, agricultural practices, and postharvest handling.

Preparing teas or infusions from the leaves is a common tradition in managing diabetes mellitus. In addition, the stems and roots can be utilized to brew infusions [12]. Some other customary applications encompass treating kidney stones, hemorrhoids, and eye ailments, as well as enhancing blood flow, lung decongestant effects promoting lactation, enhanced memory and brain function, and lowering cholesterol levels. In some laboratory experiments, chaya has demonstrated potential in terms of hypoglycemic and anti-diabetic properties. Doses and extraction methods vary from study to study, and most of the plants used are related to folk practices, since it is necessary to systematically explore extraction methods, concentrations, and doses. The present review identifies doses and extract concentrations.

Traditionally, chaya infusions were produced using water as a solvent. However, it was noted that some compounds were not fully extracted with water alone; therefore, other solvents, such as methanol and ethanol—and, in some cases, acetone—were used to improve the extraction efficiency. Studies have primarily examined the impacts of solvent extracts of chaya leaves, with findings showing that adding chaya leaves to the diets of rats had the effect of protecting the brain by reducing acetylcholinesterase levels, according to one study [21]. Furthermore, the capacity to lower blood sugar levels, as well as cholesterol and triglyceride levels, has been studied. Ethanolic extracts from chaya leaves have demonstrated promising results [22]. A recent study [19] revealed that ethanolic chaya extracts and ascorbate conferred protection against kidney and testicular damage induced by dimethylnitrosamine. Moreover, chaya shows antioxidant properties, along with inflammatory and cardioprotective effects, due to the presence of sterols, flavonoids, coumarins, and saponins [11].

Moreover, umami plays a role in developing food products by offering a rich and savory taste that improves overall food appeal. This characteristic flavor is primarily due to the presence of free amino acids, such as glutamic acid and aspartic acid, as well as 5′-nucleotides, such as 5′-AMP and 5′-GMP, along with 5′-inosine monophosphate. A research study has pinpointed acids, 5′-AMP, and short peptides as the umami components found in chaya leaves. Additionally, besides their umami flavor, chaya leaves are recognized for their medicinal properties, offering benefits for conditions such as diabetes and rheumatism, as well as gastrointestinal disorders and inflammation [23].

## 3. Phytochemistry of the Species *Cnidoscolus*

From a chemical perspective, the leaves and stems of this species have been identified to contain proteins and vitamins such as thiamine, niacin, riboflavin, retinol, beta-carotene, and ascorbic acid. It also contains minerals like calcium, iron, phosphorus, potassium, magnesium, sodium, manganese, zinc, and copper. Due to the presence of essential amino acids, the species is considered to have high nutritional value (the essential amino acids present in chaya are building blocks for protein synthesis in the body). Proteins are essential for the growth and repair of tissues, including muscles, skin, and organs [23].

The leaves contain essential oils and fatty acids like palmitic, oleic, stearic, myristic, arachidonic, and lauric acids, as well as flavones, including quercetin and kaempferol, among others like amentoflavone and astragalin (kaempferol-3-O-rutinoside). Moreover, coumarin, naringin, rutin, catechin, protocatechuic acid, and dihydromyricetin are also present in the leaves. Numerous harmful secondary substances have been identified, including glycosides like linamarin and compounds such as tannins, saponins, and lignin, which are typically removed through cooking and do not represent a safety concern [23].

Various investigations have explored the composition of *C. chayamansa* leaves through high-performance liquid chromatography (HPLC). It was discovered that the leaves harbored protocatechuic acid at a concentration of 0.242 mg/g and rutin at 2 mg/g by comparing them to known standards [24]. Chlorogenic acid in fresh leaves amounted to 1217.3 meq/kg, decreasing to 733.5 meq/kg in cooked leaves. Quercetin and kaempferol levels were measured at 44.7 µg/g and 22.4 µg/g, respectively, in raw leaves, and they decreased slightly in cooked leaves to 40.2 µg/g and 18.1 µg/g, respectively. Other identified compounds included kaempferol-3-O-rhamnosylglucoside, kaempferol-3-O-rhamnosylgalactoside-7-O-rhamnoside, and quercetin-3-O-rhamnoside in different concentrations [25].

Methanolic leaf extracts were analyzed for phenolic content using the Folin–Ciocalteu method. It was found that gallic acid (71.3 ± 1.7 mg/g), catechin (42.6 ± 3.7 mg/g), protocatechuic acid (0.24 mg/g), and rutin (2.0 mg/g) were present. Three organic extracts of the leaves were evaluated, identifying flavonoids and coumarins in the ethyl acetate extract; flavonoids, coumarins, and lignins in the ethanolic extract; and various fatty acids in the hexane extract, such as lauric acid (31.9%), myristic acid (11.7%), palmitic acid (28.3%), oleic acid (5%), and arachidonic acid (4.8%) [11,24]. The content of linamarin in the leaves and petioles of *Cnidoscolus chayamansa*, with evaluations of frozen and fresh samples, was higher in frozen samples (11.90 ± 1.8 µg/g in leaves and 14.79 ± 0.9 µg/g in petioles) than in fresh samples (2.89 ± 0.1 µg/g in leaves and 3.72 ± 1.5 µg/g in petioles). Similar results were obtained with UV–vis spectrometry, showing concentrations of 1.28 ± 0.4 µg/g in fresh leaves and 2.63 ± 0.4 µg/g in petioles, compared to 4.93 ± 2.3 µg/g in frozen leaves and 6.25 ± 0.2 µg/g in frozen petioles. The increase in linamarin in frozen plants is attributed to a physiological response to cold, which causes structural changes in lipid membranes and protein synthesis. Regarding hydrogen cyanide (HCN), the concentration in methanolic extracts determined by acid/base titration was 2.37 mg/100 g of dry extract by acid titration and 4.25 mg/100 g by alkaline titration. These levels are well below the FDA limit (20 mg HCN/100 g), indicating that chaya is safe for consumption after a five-minute cooking process. The HCN levels in chaya are considerably lower than those reported in broad beans and cassava, which range between 200 and 300 mg HCN/100 g [26]. Chaya, valued for its high content of proteins and polyphenols, was evaluated in its leaves and protein concentrates through SDS–gel electrophoresis, FTIR, and DSC. Amino acids, protein efficiency, and bioavailability were analyzed, finding protein purity of 63.52%. The identified polyphenols included anthocyanidins, hydroxycinnamic acids, hydroxybenzoic acids, and flavonols, suggesting that chaya may serve as a potential alternative source for functional foods [27].

In a study analyzing the chemical constituents of chaya leaves, samples were desiccated using both shade-drying and oven-drying techniques. Oven drying was found to be more efficient in minimizing residual moisture. The extraction of compounds was conducted through maceration using petroleum ether, ethanol, and distilled water. An examination of the constituents was executed via Soxhlet extraction processes, revealing the presence of flavonoids, alkaloids, tannins, and saponins. The moisture and total ash levels (10.7%) were within the established parameters, validating the quality of the plant [28].

## 4. Diabetes

Diabetes mellitus is a chronic condition caused by the body’s resistance to insulin, which leads to disturbances in maintaining glucose balance and can result in blood sugar fluctuations and complications related to blood vessels. The prevalence of diabetes and metabolic diseases is rapidly increasing worldwide, becoming a significant health issue with associated personal, social, and economic burdens. Data from the International Diabetes Federation indicate that an estimated 463 million adults globally are affected by diabetes. If no measures are taken to address this pandemic, projections suggest that around 578 million individuals could be dealing with diabetes by the year 2030, and this number might increase even further to 700 million by 2045. The yearly worldwide healthcare spending related to diabetes has been approximated at USD 760 billion. Therefore, the projected direct costs associated with managing diabetes are expected to reach USD 825 billion (USD 2500 per person in the US) by the year 2030 and further rise to USD 845 billion (around USD 2600 per person in the US) by the year 2045 [29].

The widespread occurrence and diverse causes of diabetes emphasize the importance of finding treatments that can address its nature and associated complications effectively. Phytotherapeutic agents from natural sources, including chaya, are increasingly preferred over synthetic drugs because of their chemical diversity and lower incidence of adverse effects [30]. Assessments of plant-based sources have shown that certain natural substances directly enhance insulin secretion, prevent apoptosis in pancreatic beta cells, and modulate the differentiation and proliferation of pancreatic beta cells. Consequently, bioactive natural compounds offer significant potential as sources of new medications [31].

### 4.1. Utilization in Diabetes

#### 4.1.1. Mechanisms of Action

The mechanisms of action of chaya against diabetes appear to be linked to compounds that influence the regulation of blood glucose levels. According to research findings, chaya extracts (especially methanolic and aqueous extracts) contain flavonoids like quercetin and rutin that could potentially lower blood sugar levels. The presence of antioxidants in chaya may aid in boosting insulin sensitivity for the management of blood sugar levels. Oxidative stress reduction by antioxidants could potentially enhance the performance of beta cells in the pancreas in terms of insulin production [24].

In diabetes, especially type 2 diabetes, cells undergo changes in how they process glucose, which may result in the production of oxygen species (ROS). These substances can harm cells by causing damage, including among beta cells that produce insulin, and can worsen insulin malfunction and resistance to it. Flavonoids are compounds found in chaya leaves and various plants, and they possess antioxidant qualities that enable them to counteract ROS’ effects by reducing oxidative stress and safeguarding cells from harm. This is crucial for diabetic cells, as it helps to maintain cellular function and protects against diabetic complications. In diabetes, especially when poorly controlled, LDL levels may be elevated. Circulating LDL can undergo oxidation due to high ROS concentrations, becoming oxidized LDL (oxLDL), which is particularly harmful. oxLDL contributes to the development of atherosclerosis, a condition where the arteries harden and narrow due to cholesterol plaque buildup, increasing the risk of cardiovascular diseases. The consumption of flavonoids, such as those present in chaya leaves, can offer significant benefits for individuals with diabetes by reducing oxidative stress, improving cardiovascular health, and protecting cells from damage. These combined effects may aid in better diabetes management and reduce the risk of associated complications [24].

An increase in LDL levels in the bloodstream poses a risk of artery disease, especially when it comes to oxidized LDL cholesterol. The “oxidative hypothesis of atherogenesis” suggests that the development of atheroma begins with foam cells generated from macrophages absorbing oxidized LDL uncontrollably within the subendothelium. These lipoproteins are harmful to the endothelium since they attract macrophages and monocytes to accumulate in the layer of blood vessels. This accumulation contributes to diseases like artery disease and is closely linked to diabetes, significantly heightening the risk of such conditions [24].

Methanolic extracts from chaya have been found to boost the activity of beta cells and enhance insulin secretion, helping to regulate blood glucose levels in rats with diabetes induced by streptozotocin (STZ). However, the mechanism does not resemble insulin’s action. Rather, it encourages efficient beta cell function. Oxidative stress is implicated in the pathogenesis of diabetes-related complications, including neuropathy, nephropathy, and retinopathy; nevertheless, the antioxidant compounds found in chaya may mitigate oxidative damage and thereby contribute to the prevention or delay of these complications [24].

**Figure 2:**
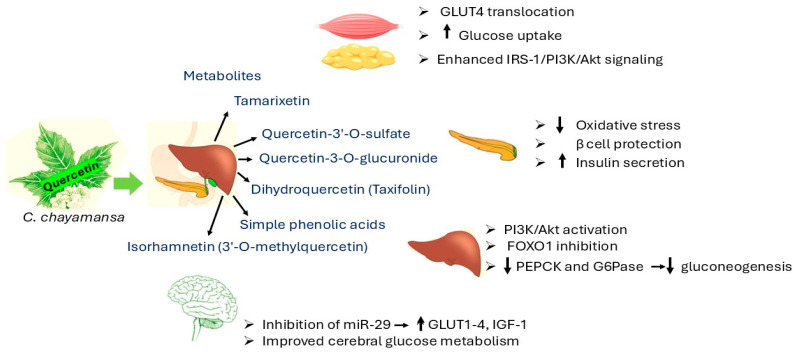
Mechanism of action of quercetin [34,35,36,37,38,39]. Akt = protein kinase B; FOXO1 = Forkhead box protein O1; G6Pase = glucose-6-phosphatase; GLUT1–4 = glucose transporter 1–4; GLUT 4 = glucose transporter type 4; IGF-1 = insulin-like growth factor-1; IRS-1 = insulin receptor substrate 1; miR-29 = microRNA-29; PEPCK = phosphoenolpyruvate carboxykinase; PI3K = phosphoinositide 3-kinase.

The plant’s effectiveness in lowering blood sugar levels through activity seems to depend on the concentration of the extract being used, according to studies conducted with different doses, which revealed similar outcomes to commonly prescribed medications like metformin and glibenclamide [32]. When tested on rats in lab settings, chaya methanolic extracts showed a decrease in blood glucose levels after glucose intake, which hints at its potential in managing post-meal blood sugar spikes [33]. The bioactive components found in the extract, including quercetin and rutin, are believed to be responsible for these properties. These substances could enhance the body’s response to insulin and have antioxidant effects, offering properties that support the management of diabetes [26]. Figure 2 represents mechanism of action of quercetin.

In addition to reducing glucose levels, chaya extracts have also been shown to reduce cholesterol and triglyceride levels in animal models, contributing to improved overall metabolic health in diabetic conditions. Type 2 diabetes often leads to inflammation and insulin resistance issues among affected individuals; the antioxidants and anti-inflammatory properties found in chaya may aid in reducing inflammation, according to research [32]. Overall, the anti-diabetic activity of chaya leaves is attributed to the combined action of its bioactive compounds, which enhance insulin secretion, reduce postprandial glucose, and have antioxidant and lipid profile improvement effects. These functions position chaya as a supplementary treatment choice for the management of diabetes.

#### 4.1.2. In Vivo Studies

Several in vivo studies have explored how methanolic (MeOH) extracts of *Cnidoscolus chayamansa* affect blood sugar levels in Wistar rats with diabetes induced by streptozotocin (STZ). Wistar rats with streptozotocin (STZ)-induced diabetes were administered intraperitoneal doses of 10, 40, and 70 mg/kg, which were subsequently evaluated through postprandial glucose testing and fasting blood glucose measurements. It was discovered that only the 70 mg dose (twice the weight of a grain of rice) had an impact in lowering blood sugar levels when compared to the glibenclamide-treated group. There was a 1% decrease at 120 min (about 4 h) and an 18% decrease at 180 min (6 h). Nevertheless, this dosage did not exhibit a fasting effect, as seen with insulin [24,33].

Another study evaluated the effects of methanolic (MeOH) extracts in rats with alloxan-induced diabetes by administering doses of 0.5, 1.0, and 1.5 mg/kg. The outcomes revealed the levels of glucose, cholesterol, and triglycerides, with the reduction in glucose correlating to the dosage used. These results were comparable to the effects observed with standard anti-diabetic drugs such as metformin and glibenclamide [32].

Furthermore, experiments conducted in rats using water extracts from dried *Cnidoscolust chayamansa* leaves revealed a decrease in both body weight and blood sugar levels, which was linked to the presence of quercetin and rutin, suggesting the extract’s potential efficacy and safety for consumption [26].

Recent studies have revealed that extracts from *C. chayamansa*, *E. prostrata*, and *J. dioica* contain ingredients known for their antioxidant properties. In this investigation, the amounts of these components were determined to be 6.34, 10.67, and 1.83 mg gallic acid equivalents/g of dry biomass (DB), respectively. Water-soluble antioxidants were measured using the ABTS method, yielding 5.89, 12.70, and 2.50 millimoles of Trolox equivalents/g of DB. After examining tissues under a microscope following the administration of these extracts during hyperglycemia induction, they showed changes that implied a beneficial effect [40].

Another study examined the impacts of aqueous extracts of chaya on glycemic control in streptozotocin-induced diabetic rat models. Chaya extracts from plants collected in Quintana Roo (Mayan region) and Durango (Northern Mexico) were compared, with metformin used as a reference drug for control purposes. The compositions of the extracts from both areas were examined using high-performance liquid chromatography coupled with tandem mass spectrometry (HPLC-MS/MS-QQQ). The researchers conducted this study on tissues to investigate the impacts of extracts on Langerhans islets. At two weeks of treatment, with both extracts, hypoglycemic effects were evidenced. There was partial recovery of Langerhans islets. This effect of the treatment was consistent despite the plants being grown 2350 km apart under varying conditions. The compounds found in the two extracts showed no differences [41].

In another research study performed on type 2 diabetic rats, it was examined how combining metformin with green tea and black tea derived from *Cnidoscolous aconitifolius* impacted the rats’ health over a period of 28 days (around 4 weeks). The levels of phenols and flavonoids, as well as the antioxidant capacities of the teas, were analyzed. The study also explored blood sugar levels after fasting, as well as lipid profiles and markers related to liver and kidney function. Green tea exhibited the highest levels of phenols and flavonoids and demonstrated superior antioxidant properties. When combined with metformin, in a study group of participants who consumed both teas together with the medication, it showed improvements in health markers including fasting glucose levels and various blood lipid parameters, such as triglycerides and cholesterol levels (total and LDL). Additionally, a reduction in liver enzymes—alanine aminotransferase (ALT) and aspartate aminotransferase (AST)—as well as in creatinine and urea levels was observed, alongside an increase in serum protein levels. The combination of chaya tea infusions with metformin was more effective than metformin alone in regulating fasting glucose, with green tea being the most effective [42].

Another study focused on assessing the protective effects of chaya leaf on mitochondrial abnormalities and synaptic damage in the TallyHO (TH) mouse model of type 2 diabetes, which shares several characteristics with human diabetes. The results showed that diabetic TH mice had body weight and fasting glucose levels comparable to those of diabetic mice, but those fed chaya experienced notable reductions in both areas. This information suggests that chaya may provide a safeguard against mitochondrial and synaptic dysfunction in mice. This implies that chaya could be beneficial as a supplement for people at risk of or dealing with diabetes and metabolic disorders [43].

In a research study investigating the effects of methanolic leaf extracts of *Cnidoscolus aconitifolius* in alloxan-induced diabetic rats, GC-MS analysis was conducted as part of the experimental evaluation. The analysis revealed the presence of 37 compounds, including hydrocarbons, alcohols, phenols, and fatty acids, many of which possess biological activity. Different doses of the extract (100 mg/kg, 150 mg/kg, and 200 mg/kg) were administered to diabetic rats, resulting in a reduction in blood sugar levels, outperforming chlorpropamide, a known diabetes medication. These findings suggest that the plant may help in controlling blood sugar levels in people with diabetes [44].

Type 2 diabetes is a health concern that affects many people globally. While there are medications to manage blood sugar levels orally, some individuals opt for herbal remedies due to their proven benefits. However, limited research has been conducted to investigate the interactions between medications like metformin and herbal remedies. These interactions could potentially impact the effectiveness of each treatment. In a study, the combination of metformin and chaya (an extract of *Cnidoscolus aconitifolius*) was examined in Long Evans rats with diabetes induced by streptozotocin. Various combinations were tested, and blood glucose levels were measured in the rats. The compositions of the extracts were analyzed using HPLC-MS/MS. The results showed an antagonistic effect between the compounds, with high glucose levels maintained in three of the four treated groups. Certain identified elements present in chaya could explain this phenomenon. In conclusion, the findings suggest that the consistent consumption of chaya as a treatment might diminish the efficacy of metformin, as observed in part of the Mexican population. It is advisable to conduct investigations on pharmacodynamic interactions to better understand this behavior [45].

Patients diagnosed with type 2 diabetes commonly face inflammation caused by the production of pro-inflammatory proteins, like TNF alpha and ILS, due to NF-kB regulation and overexpression. A study aimed to explore the potential anti-inflammatory properties of specific fractions of chaya aqueous extracts that have been previously suggested to reduce inflammation levels in diabetic patients. The researchers examined the content of the sample fractions by employing solid-phase extraction and tracking them with HPLC-MS/MS analysis. The activation of NF-kB and the levels of the pro-inflammatory proteins TNF-alpha and IL-6 were evaluated in streptozotocin-induced diabetic rats. Two of the sample fractions exhibited notable anti-inflammatory properties in diabetic rats induced by streptozotocin. Two of the fractions demonstrated the most significant anti-inflammatory effects, even outperforming the crude extract, which contained the highest concentrations of compounds. The presence of ferulic acid in the chemical profile suggested that it may have played a key role in the observed anti-inflammatory effect. On the other hand, naringenin and apigenin, found exclusively in the extract, might have impeded this effect, accounting for the superior performance of these distinct fractions [46]. Table 1 summarizes chaya’s biological activity, extract types, and dosages

#### 4.1.3. In Vitro Studies

In a study performed to assess the antioxidant properties of a methanolic extract of *Cnidoscolus chayamansa* using a colorimetric method, when tested at a concentration of 5 mg/mL, the DPPH radical was inhibited by 45%. The ABTS radical showed an inhibition rate of 94%. At a concentration of 1 mg/mL, the inhibitory effects decreased to 13.75% for DPPH and 52.07% for the ABTS radical. The sample displayed antioxidant effectiveness (IC50 = 1693 µg/mL) in contrast to positive controls (gallic acid and Trolox), with IC50 values of 15.39 and 43.90 µg/mL, respectively [24].

Another assessment of the antioxidant properties through the oxygen absorbance capacity (ORAC) method found that uncooked leaves exhibited an ORAC value of 15.6 µeq Trolox/g. Meanwhile, cooked leaves showed a slightly lower value of 14.8 µeq Trolox/g. Furthermore, the total amount of phenolic compounds present was determined to be 1217.3 meq chlorogenic acid/kg in raw leaves and 733.5 meq/kg in cooked leaves. The decrease of 5% in ORAC and the 40% decrease in phenols in the leaves were attributed to the change in quercetin-3-O-rhamnoside content, which increased by 23% in the cooked leaves [25].

Moreover, when assessing the antioxidant capacity with the DPPH method across three leaf extracts, it was observed that the hexane extract saw a reduction of 10.58%, while that of the ethyl acetate extract decreased by 11.68%. The ethanol extract exhibited a decline of 10.66%. The ethanol extract displayed polyphenol content of 35.70 meq AG/g according to the Folin–Ciocalteu method. This was followed by the hexane extract at 22.35 meq AG/g and the acetic ether extract at 13.25 meq AG/g.

Another way to evaluate the antioxidant capacity in laboratory settings involves using the ferric reducing ability of plasma (FRAP) method. In this method, the hexane extract (Hex) showed an inhibition rate of 239.47 µmol Fe^+2^/L, the ethyl acetate extract (AcOEt) showed an inhibition rate of 387 µmol Fe^+2^/L, and the ethanolic extract (EtOH) showed an inhibition rate of 254 µmol Fe^+2^/L [11]. According to [47], this method provides insights into the antioxidant properties. This study found that a 95% ethanolic extract of *C. chayamansa* showed antioxidant properties in tests using DPPH and FRAP micromethods. However, when tested using the macromethod approach, the antioxidant effect was minimal, leading to the conclusion that the EtOH extract may not exhibit antioxidant properties [47].

Non-communicable chronic diseases (NCDs) are the leading causes of death worldwide. They are associated with oxidative stress and inflammation. Researchers have found that certain natural compounds derived from plants like *Cnidoscolus aconitifolius* could play a role in preventing these diseases by helping to combat stress and inflammation. In another in vitro study, the antioxidant and anti-inflammatory activity of *C. aconitifolius* extracts were evaluated, highlighting their notable potential due to the presence of bioactive compounds. These findings hold implications for diseases like diabetes, a type of NCD, where oxidative stress and inflammation play roles in disease progression and management. Chaya extracts may have advantages in the treatment of diabetes by alleviating these detrimental processes [48].

A different research project focused on creating and assessing capsules containing dried *Cnidoscolus chayamansa* McVaugh extract through hot percolation extraction using a 70% *v/v* ethanol solution. Preformulation experiments were carried out to prepare three sets of capsules. Various properties, like the moisture level, drug content, and disintegration weight consistency, as well as the dissolution of the capsules, were analyzed. One formulation showed optimal drug release in in vitro tests and met the standards of the Pharmacopeia. It was concluded that these capsules are an effective means of administering *C. chayamansa* extracts for the management of diabetes mellitus [49].

Another study aimed to evaluate the antioxidant capacity of a water-based extract and a combined extract (methanol/acetone/water) derived from chaya leaves and their impacts on mitochondrial bioenergetics and fatty acid oxidation. The antioxidant properties of both extracts derived from chaya leaves were found to be similar to those of nopal, a plant commonly used in medicine. The results indicated that the liquid extract from chaya leaves improved the function of mitochondria and the oxidation of fatty acids in liver cells. This suggests that it could be a treatment for liver issues linked to metabolic disorders [50].

In addition, research conducted at the University of Querétaro in Mexico involved the cultivation of *Cnidoscolus aconitifolius* (chaya) from woody cuttings, initiated in 2014. During the summer of 2016, leaf samples were collected from all cultivated plants to analyze methanol–water and ethanol–water extracts (in 50:50 and 80:20 ratios) from both raw and boiled leaves. The objective was to evaluate the total phenolic content, total flavonoid content, and antioxidant capacity. Through RP-HPLC-DAD analysis, 11 phenolic compounds were identified and quantified, including gallic acid, vanillin, chlorogenic acid, and resveratrol. The findings revealed that boiling the leaves increased both the antioxidant capacity and phenolic compound content compared to raw leaves. The 80:20 ethanol extract showed the highest polyphenol concentration, suggesting that chaya leaves are a rich source of natural antioxidants [51].

A separate study was initially conducted on four atolls south of Tarawa in Kiribati and later extended to Tuvalu to investigate the mineral levels present in different types of local edible leafy plants. Variations in the ability of these plants to accumulate minerals in their leaves were analyzed, as well as their resistance to soils lacking essential nutrients. Farmers in the area grew plants in nurseries to then provide them to the rest of the farmers. Information leaflets highlighted the most suitable species among the 24 studied, including multiple examples of each. These included plants such as chaya, ofenga, hedgerow panax, and purslane, which have been shown in other studies to have beneficial effects against non-communicable diseases (NCDs) [18].

Chaya is a shrub known for its medicinal properties, used since the time of the Mayan culture in Southeastern Mexico, and has been studied to evaluate its effectiveness in reducing blood pressure and blood glucose levels through six different types of leaf extracts (aqueous and alcoholic). In a recent study, the leaves used were collected in Timucuy, Yucatan, Mexico as part of the process for this specific study. For the extraction, 48 h maceration was used, with the proportions consisting of one part of the sample for every ten parts of the solvent (weight/volume). The solvents used were the following: distilled water, a mixture of ethanol and acetone together with ethyl acetate, diethyl ether, and hexane. The tests carried out included enzymatic inhibition against ACE, and the inhibition effects on the alpha glucosidase and alpha amylase enzymes were also evaluated in tests under controlled conditions. The hypotensive impact, the glycemic response to oral glucose overload, and the hypoglycemic effects in rats induced with obesity, hypertension, and hyperglycemia were also analyzed. The extracts with the lowest 50% inhibition concentrations (IC50) for angiotensin-converting enzyme (ACE), alpha glucosidase, and alpha amylase were the following: acetone (12.61 µg/mL), ethyl acetate (22.97 µg/mL), and ethyl acetate (32 µg/mL). On the other hand, the aquaculture extract had a notable hypotensive effect, reducing systolic blood pressure by 15.5%. The highest percentage reduction in systolic blood pressure was observed in the methanol extract at a rate of 23%, while the hexane extract managed to reduce glucose levels by up to 22.88%. The findings revealed that all chaya extracts exhibited enzyme-inhibitory properties and hypotensive and hypoglycemic effects [52]. The use of different extracts is an advantage in studies, as it allows for the evaluation of various substances. However, the chemical profile of each extract should be determined to identify its composition and bioactive compounds. Since different articles present varying extraction methodologies, comparing their effects becomes difficult. Therefore, a study design that considers these variables is necessary to accurately assess the effects of chaya.

The study performed in [53] compared fried, roasted, boiled, and raw chaya in terms of the content of bioactives and nutrients, as well as antioxidant, anti-diabetic, and anti-thrombotic activity. It was concluded that chaya is a good option, finding that fried chaya increased the antioxidant activity and α-amylase inhibition (+30%) but that the boiled chaya decreased all parameters. Frying and roasting improved the sensory attributes while maintaining the plant’s nutritional value and enhancing its functional properties, making chaya promising for dietary interventions in diabetes.

**Table 1:** Summary of chaya’s biological activity, extract types, and dosages.

Antioxidant	Anti-Inflammatory	Cardioprotective/Hypotensive	Renal Protection	Cognitive Function	Hypoglycemic	Anti-Mutagenic	Anti-Hypercholesterolemic	Anti-Hypertriglyceridemic	In Vitro	In Vivo	Model	Type of Extract	Clinica Assay	Extract	Dose	Drug Interactions	Toxicity	Reference
X	X	X							X	X	Male CD1 mice	Hex, EtOAc, and EtOH		135 g/5.68 g Hex, 5.27 g EtOAc, 9.44 g EtOH	2 mg, 500 mg/kg	-	LD50 > 5 g/kg EtOAC and EtOH, single dose	[11]
X									X			H_2_O, EtOH		-	-	-	-	[17]
X			X							X	Wistar rats	EtOH		600 g to yield 12.78 g after evaporation to dryness	400 mg/kg	-	-	[19]
X					X				X	X	Wistar rats	H_2_O		5 g/100 mL	5 mg/kg	-	LD50 1070.42 μg/mL	[20]
				X						X	Wistar Rats	Fine powder of dried leaves as meal		-	1%, 2.5%, 5%, and 10% supplemented diet	-	-	[21]
					X		X	X		X	Mice	EtOH, Hex, MeOH, Chl		5000/2.5 L EtOH	250, 500, 1000, 2500, 3000, 3500, 4000, 4500, and 5000 mg/kg	-	EtOH LD50 of 4000 mg/kg, MeOH LD50 3500 mg/kg, Hex LD50 value of 2000 mg/kg, Chl LD50 2500 mg/kg	[22]
X					X	X			X	X	Wistar rats	MeOH		500 g/L	10, 40, and 70 mg/kg	-	-	[24]
X									X			H_2_O		-	-	-	-	[25]
					X		X			X	Rats	MeOH		20 g of dried leaves in 250 mL 80% MeOH	0.5 and 1.5 g/kg	-	-	[32]
X					X				X	X	Wistar rats	H_2_O		60 g/300 H_2_O	-	-	-	[40]
					X					X	Wistar rats	H_2_O		15 g of fresh leaf/L	-	-	-	[41]
X					X		X		X	X	Wistar rats	H_2_O		2 g of tea powder (“green” or “black”) was infused (4 min) in 100 mL of boiling water	6 mL/kg	Metformin	-	[42]
X									X			EtOH		-	-	-	-	[47]
					X					X	Mice TallyHo/JngJ strain (TH) and mice SWR/J strain	Meal as powder		Diet with 25% ground chaya	-	-	-	[43]
					X					X	Wistar rats	MeOH		-	100, 150, and 200 mg/kg	-	-	[44]
					X					X	Rats	H_2_O		7.5 mg of dry leaf in 1 L	25 to 100% extract	Metformin	.	[45]
	X				X					X	Long Evans rats	H_2_O, EtOH, AcOEt		2.5 g/100 mL water	Ad libitum as drinking water	-	-	[46]
X	X								X			H_2_O, EtOH, Ace, AcOEt, Et2O, Hex		1:10 (*w*/*v*)	150, 300, 450, 600, 750, and 900 mg/mL; 10, 25, 50, 100, 250, and 500 mg/mL; 25, 50, and 100 mg/mL	-	-	[48]
X									X			EtOH-H_2_O-MeOH-H_2_O		1:10	-	-	-	[51]
		X			X				X	X	Wistar rats	H_2_O, EtOH, Ace, AcOEt, Et2O, Hex		1:10	-	-	-	[52]
X		X			X				X			H_2_O	X	5 g/100 mL	-	-	-	[53]

Abbreviations. H_2_O, water; EtOH, ethanol; Ace, acetone; AcOEt, ethyl acetate; Et2O, ethyl ether; Hex, hexane. “—“ indicates information not established. Dose: expressed in kg of body weight. “X” indicates a characteristic found in the study.

#### 4.1.4. Clinical Studies

Proper nutrition is essential in maintaining good health; to achieve this, the diet should include vegetables such as quelites. Quelites are edible wild plants that may help to prevent diabetes due to their antioxidant and anti-inflammatory compounds. A study was conducted to determine the glycemic index (GI) and glycemic load (GL) of rice and a tamal prepared with and without two types of quelites: alache (*Anoda cristata*) and chaya (*Cnidoscolus aconitifolius*). The GI was measured in 10 healthy subjects, namely seven women and three men, with the following average metrics: age, 23 years; body weight, 61.3 kg; height, 1.65 m; body mass index, 22.7 kg/m^2^; and fasting glucose, 77.4 mg/dL. Capillary blood samples were collected within 2 h after the meal. White rice (rice without quelites) had a GI of 75.35 ± 15.6 and a GL of 36.17 ± 7.8; rice with alache had a GI of 33.74 ± 5.85 and a GL of 33.74 ± 1.85. White tamal had a GI of 57.33 10.23 and a GL of 26.65 ± 5.12; tamal with chaya had a GI of 46.73 ± 22.1 and a GL of 23.36 ± 11. The GI and GL values recorded for the combinations of quelites with rice and tamal confirmed that quelites could be a beneficial alternative for healthy diets [54]. Based on in vitro studies, more clinical research is needed to establish the minimum and maximum effective doses. Until the appropriate dosing, the environmental influences on the metabolite profile of chaya, and its interaction with various drugs used in the treatment of diabetes are established, it will be difficult to position it as a suitable tool in diabetes management, and its effects will remain inconsistent. Table 2 summarizes limitations considered in researches performed in chaya.

## 5. Conclusions

In conclusion, scientific evidence supports the anti-diabetic properties of chaya leaf, highlighting its hypoglycemic and antioxidant properties. In vivo studies have demonstrated that the methanolic extract of *C. chayamansa* significantly reduces blood glucose levels in rats with streptozotocin- and alloxan-induced diabetes, favorably comparing with conventional medications such as metformin and glibenclamide [24,32,33]. Additionally, research has revealed that aqueous extracts of chaya not only decrease glucose levels but also improve lipid profiles and overall health in diabetes models, demonstrating its therapeutic potential [26,42].

Studies have also highlighted the role of the phenolic and antioxidant compounds present in chaya, such as quercetin and rutin, in reducing oxidative stress, a key factor in the pathogenesis of diabetes [40]. The combination of chaya extracts with standard treatments like metformin has shown promising results, although possible interactions have been observed, requiring further investigation to optimize their combined use [42,47].

In vitro research corroborates the antioxidant and anti-inflammatory capacity of chaya, suggesting that its consumption could mitigate the harmful effects of oxidative stress and inflammation in diabetes [48]. Additionally, the development of standardized formulations of chaya extracts, such as capsules, provides an effective means of administration that meets pharmaceutical standards, facilitating its use in diabetes management [49]; however, there is a need for evaluations to determine suitable formulations.

In summary, chaya leaf represents a valuable and safe option in complementary therapies for diabetes, underscoring the need for further clinical studies in humans to confirm and expand these findings. The integration of herbal remedies with conventional treatments could offer a more holistic and effective approach to managing this chronic disease, but it is necessary to systematically explore doses, extraction methods, concentrations, the effects of isolated compounds, and interactions with other drugs for diabetes treatment; without this information, this plant will remain in the domain of folk medicine.

## Figures and Tables

**Table 2:** Summary of the limitations considered in the studies.

Issue Assessed	Study Limitations
Experimental Lab/Animal Studies	Mainly in vitro/in vivo studies; clinical applicability limited. Animal models only, lack of human or clinical trial support. Food matrix interaction is not fully understood. Limited dose standardization—no specific dosing guidelines or adverse effects discussed. No long-term effects studied. No correlation with human nutritional outcomes. High heterogeneity in methods; limited clinical correlation. Differences in animal models and impairment in sex distribution and variance because of species.
Ethnobotanical	Mostly descriptive. Biological validation of plant evaluated. Does not link genetics and environmental changes to bioactivity.
Phytochemical/Nutritional Studies	Lacks bioavailability and toxicity data; no in vivo relevance. High heterogeneity in methods; limited clinical correlation.

## Data Availability

The data presented in this study are available on request from the corresponding authors.
